# Adult-Onset Neuroepidemiology in Finland: Lessons to Learn and Work to Do

**DOI:** 10.3390/jcm12123972

**Published:** 2023-06-11

**Authors:** Jussi O. T. Sipilä

**Affiliations:** 1 Department of Neurology, North Karelia Central Hospital, Siun Sote, 80210 Joensuu, Finland; jussi.sipila@utu.fi; 2 Clinical Neurosciences, Faculty of Medicine, University of Turku, 20014 Turku, Finland

**Keywords:** epidemiology, incidence, prevalence, regional differences, registry

## Abstract

Finland is a relatively small genetic isolate with a genetically non-homogenous population. Available Finnish data on neuroepidemiology of adult-onset disorders are limited, and this paper describes the conclusions that can be drawn and their implications. Apparently, Finnish people have a (relatively) high risk of developing Unverricht-Lundborg disease (EPM1), Multiple Sclerosis (MS), Amyotrophic Lateral Sclerosis (ALS), Spinal muscular atrophy, Jokela type (SMAJ) and adult-onset dystonia. On the other hand, some disorders, such as Friedreich’s ataxia (FRDA) and Wilson’s disease (WD), are almost absent or completely absent in the population. Valid and timely data concerning even many common disorders, such as stroke, migraine, neuropathy, Alzheimer’s disease and Parkinson’s disease, are unavailable, and there are virtually no data on many less-common neurological disorders, such as neurosarcoidosis or autoimmune encephalitides. There also appear to be marked regional differences in the incidence and prevalence of many diseases, suggesting that non-granular nationwide data may be misleading in many cases. Concentrated efforts to advance neuroepidemiological research in the country would be of clinical, administrative and scientific benefit, but currently, all progress is blocked by administrative and financial obstacles.

## 1. Introduction

**Figure 1:**
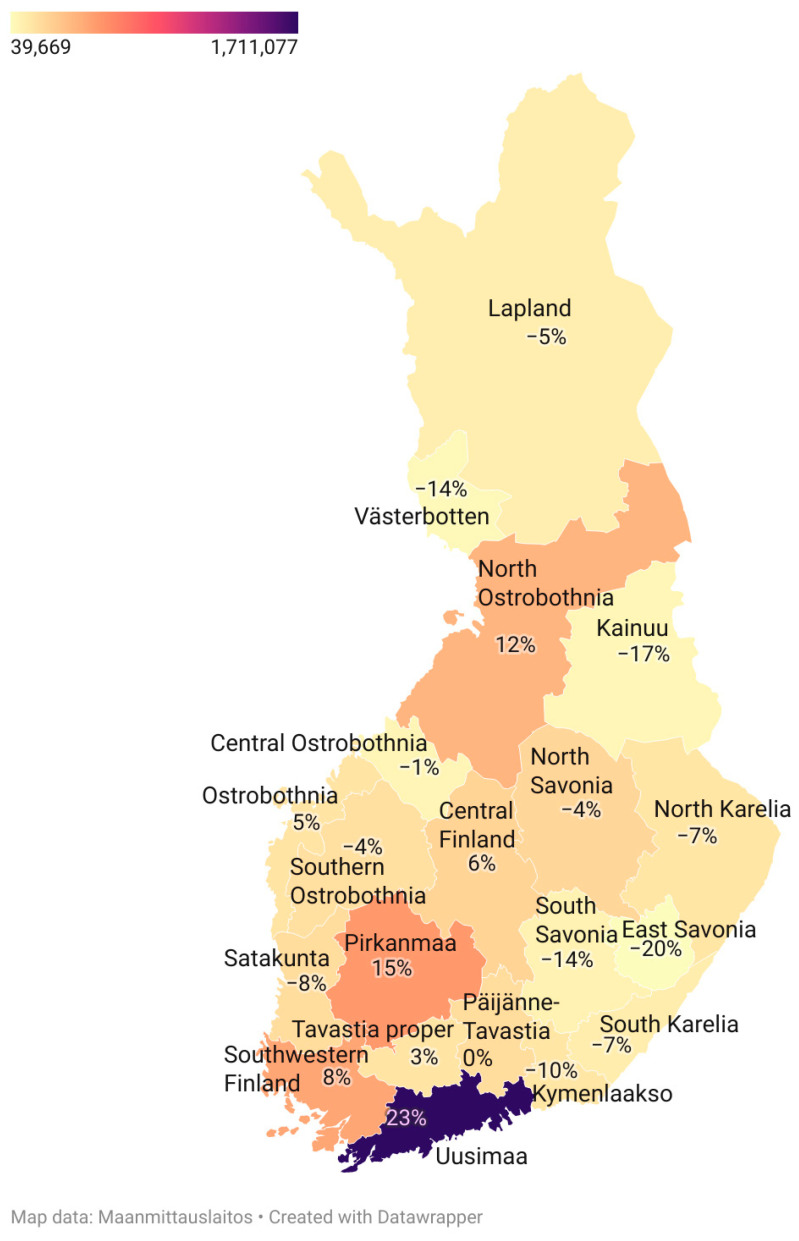
The regional total population count (the colours) at the end of the year 2021 and the change in them (%) compared to the year, 2000.

Finland is a relatively sparsely inhabited country, with a total population of slightly over 5.5 million (Figure 1), and it is within the genetic margins of Europe [1,2,3]. Its genetic history is marked by bottlenecks, founder effects and drift [4,5,6]. A well-known result of this is the Finnish Disease Heritage (FDH): 39 monogenic diseases are more much more common in Finland than they are anywhere else [7]. Probably resulting from migration history in the 16th century, these disorders are more common in the northern and eastern parts of the country. This pattern is also rather similar to the observed autosomal substructure of the Finnish population [8,9]. Indeed, western and eastern Finnish people are more genetically distant from each other than the English are from the Germans [10]. This is apparently a result of male-dominant migration influx to western parts of the country [11,12]. Although the population became genetically more heterogenous after the Second World War, the rate of change differed considerably by region, and the population in the western parts of the country remains generally healthier and wealthier than those in the eastern and northern parts, probably partly because of cultural, societal and environmental factors [13,14,15,16]. Consequently, it is nowadays sometimes stated in the media that there are ”two Finlands”.

Although that is an exacerbation, it is certainly true that the Finnish population is not homogenous regarding medical and epidemiological considerations. Clinicians in different parts of Finland may face surprisingly different numbers of the same diseases, and studies that aggregate epidemiological data at the national level without regional subanalyses may obscure important details and trends. On the other hand, high-quality administrative and health archives in the country present a unique study opportunity. Some adult-onset diseases of the nervous system serve as examples here.

## 2. Nuances of Rarity

The FDH includes diseases that are more common in Finland than they are in other parts of the world [7]. Among adult neurological patients, the most common disease is Unverricht-Lundborg disease (progressive myoclonic epilepsy type 1, EPM1). However, with a world-record-age-standardised point prevalence of 1.53/100,000 in 2016 [17], the disease is was clearly less common in the country than another rare neurodegenerative disorder is, Huntington’s disease (HD), the point prevalence of which was 2.09/100,000 persons in 2010 [18], and it appears to be limited to 1.5–2.5/100,000 by population chromosome 4 haplotype distribution [19]. This is naturally partly explained by the differing mode of inheritance (autosomal dominant in HD vs. autosomal recessive in EPM1). Nevertheless, considering that HD is 4–5-fold more common in other Caucasian populations [20], the difference highlights the need for specificity, clarity and regional considerations when discussing rare diseases. While HD is spread rather evenly in the population across the country, EPM1 appears somewhat more common in the east than it is the west and all but absent from the northernmost hospital districts.

On the other hand, there are neurological diseases that are quite common elsewhere, but practically absent in Finland. The most obvious examples probably are Friedreich’s ataxia (FRDA) and Spinocerebellar ataxia type 3 (SCA3) [21,22]. On the other hand, SCAs 7 and 8 seem to be over-represented [20,22,23]. The frequency of mitochondrial DNA polymerase gamma (POLG) mutations, which lead to mitochondrial recessive ataxia syndrome (MIRAS), is also relatively high [24,25,26], Moreover, although Fragile X–Associated Tremor/Ataxia Syndrome (FXTAS) has been reported in Finland [24,27], no epidemiological data are available. The frequency of FMR1 mutations in Finland is unknown, but considering previous data [28,29], it does not appear to be uncommon. Similarly, RFC1 also seems to be a part of the list of causes of ataxia in the Finnish population [24,30], and although the author can disclose that the current prevalence of diagnosed RFC1 disease (all clinically manifest phenotypes) in North Karelia is 5.5/100,000, more robust epidemiological data are needed. There are some singularly identifiable pathogenic mutations in other genes at well [21,24,31]. No data are available on epidemiology of non-genetic ataxias in Finland. Summa summarum, international data and guidelines of genetic testing and treatments for ataxia are of very limited value to Finnish clinicians treating patients with potentially heritable ataxias. However, as some causes may be found more often here than they are found elsewhere, treatment trials for these disorders might be especially feasible in Finland. A comprehensive, nationwide study of hereditary causes of adult-onset ataxia in (different parts of) Finland is clearly needed, preferably also leading to a clinical registry to enable recruitment to clinical trials. The lessons learned from the process of building an HD registry in Finland may help with this [32]. Furthermore, it should also be noted that the available data on the global epidemiology of ataxias are quite patchy [33]. Similar endeavours would, therefore, be welcome around the world.

Another example of a rare disease that is almost absent in Finland is Wilson’s disease (WD): there were only 25 living persons with diagnosed WD in Finland in 2017, indicating a point prevalence of 0.45/100,000 persons. Moreover, a third of the patients were immigrants, indicating that the disease is very rare among native Finnish people [34]. Almost 50% of the 17 new diagnoses recorded during the 1998–2017 study period had been made in two hospital districts (south-western Finland and northern Ostrobothnia, which are together inhabited by 16% of the population), indicating that the disease is extremely rare in most of the country. Therefore, and because the patients are taken care of by paediatricians and gastroenterologists, adult neurologists extremely seldom see WD patients in Finland. Interestingly, recent data concerning genotype–phenotype correlations in Finnish WD patients suggest that these characteristics that have not been observed in larger European patient cohorts [35]. This needs to be verified in larger cohorts, but it also suggests that investigations of even small patient populations may provide unique insights.

## 3. High Risk and Even Higher Risk

**Figure 2:**
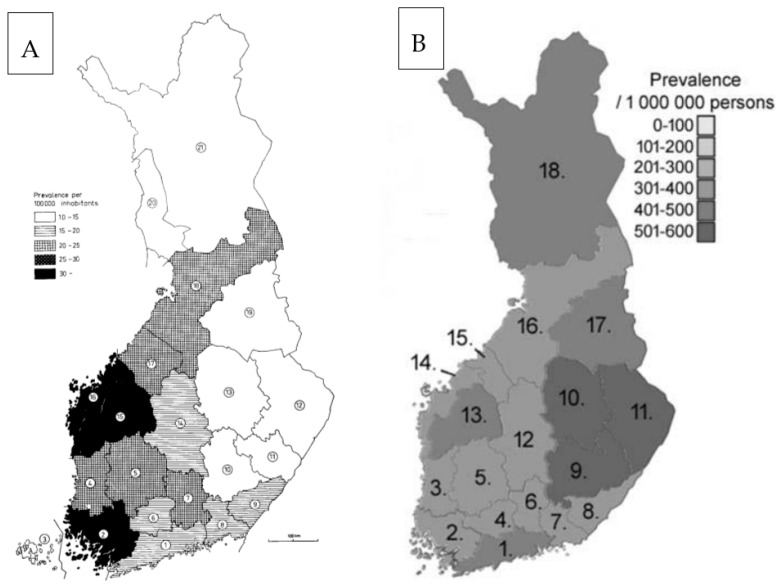
(**A**) The regional prevalence of MS in Finland in 1964 [55]. (**B**) Prevalence distribution (shaded colour) of adult-onset dystonia in Finland at the end of the year 2016 [56]. The numbers within the maps point to the names of the regions in the original publications, lists of which are not provided here (please refer to Figure 1 for the names).

Finland’s population is also at high risk of developing some neurological diseases. Multiple Sclerosis (MS) is the most documented one of these [36,37,38]. Certain areas in Finland are among those with the highest reported MS risk in the world. However, similarly to the Norwegian data, the Finnish reports also contest the reported global latitudinal MS gradient, indicating that more fine-grained epidemiological data are needed [38,39]. Interestingly, regions with the highest MS incidence and prevalence in Finland are located in the western parts of the country, but they are not immediately on the coast (Figure 2) [38]. The causes of this distribution are unknown, but many ideas have been put forward, including, i.e., geochemical factors [40,41], but these do not seem to be epidemiologically optimal [42]. Furthermore, the easternmost part of the country also shows an equal MS incidence between men and women, although this may be at least partially related to demographic factors [43]. Nevertheless, these regional differences suggest that a thorough comparison of decades-long time trends of MS incidence in different Nordic areas has the potential to unveil new insights into MS pathogenesis [44]. This would be all the more pertinent since there is still a considerable amount of uncertainty about whether MS incidence has increased over time and, on the other hand, there are data that even suggest that it might have declined at least in some areas more recently [45,46,47]. Of note, there are no data to suggest that Finland’s population has an overall high risk of developing neuroimmunological disorders. The incidence of Guillain-Barré syndrome (GBS) and Neuromyelitis Optica (NMO) appear to be largely similar to those of other European populations [48,49,50,51]. Much more data are, however, clearly needed on other neuroimmunological disorders besides MS before any firm conclusions can be made, especially considering the high risk of other autoimmune disorders, such as type 1 diabetes and rheumatoid arthritis, in the country [52,53,54].

**Figure 3:**
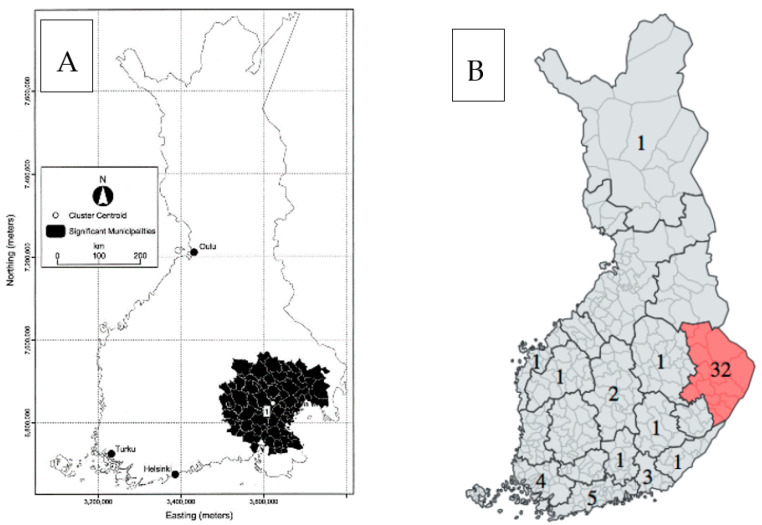
(**A**) The amytotrophic sclerosis hotspot (by birthplace) of Finland [60]. (**B**) The number of Spinal muscular atrophy Jokela-type patients per region in Finland identified during Manu Jokela’s thesis project [67].

Finland has also been identified as a high-risk area for Amyotrophic Lateral Sclerosis (ALS) [57,58,59]. Regional data are scarce, but the disease appears to be fairly evenly distributed across the country, albeit with a hotspot in the south-eastern parts of the country (Figure 3) [59,60]. C9orf72 and SOD1 mutations are the most common genetic causes, and they probably contribute to the high incidence of ALS in Finland [61]. Recent data also suggest regional differences (C9orf72 hexanucleotide repeat expansion is found more often in the east, and SOD1 mutations are found more often in south-western Finland) [59,62]. This probably also explains why the standardized (per the 2013 European Standard Population) incidence of Frontotemporal Lobar Degeneration (FTD) is so high in northern Savo (7.01/100,000) and lower (4.16/100,000), although still relatively high (the estimated annual incidence rate in Europe being 2.36/100,000), in northern Ostrobothnia [63]. Overlapping just north of the ALS hotspot, north Karelia (Figure 3) is an eastern high-incidence area for another motor neuron disorder that must be considered in the differential diagnosis of ALS, namely Spinal muscular atrophy, Jokela type (SMAJ, previously called Late-Onset Spinal Motor Neuronopathy or LOSMoN) [64,65,66]. SMAJ is caused by a dominant mutation in CHCHD10, and before it was identified, these cases were probably diagnosed as slowly progressing ALS and confounded epidemiological studies. Making the correct diagnosis between these two is particularly important in light of the considerable difference in their prognoses. Data on the global SMAJ prevalence are scarce, indicating that further research on the epidemiology of motor neuron disorders are needed.

Adult-onset dystonia appears to be as prevalent in the southern provinces of Uusimaa and Pirkanmaa as it does in other European countries, although cervical dystonia seems more common [68]. However, the disease is much more common in the eastern provinces of north Savonia and south Savonia, along with north Karelia, where the prevalence is the highest in the country (526/100,000), with almost a third more than the number of cases in Pirkanmaa [56]. Unfortunately, no data on adult-onset dystonia incidence are available. Interestingly, the regional prevalence distribution of dystonia in Finland looks almost like a mirror-image of that of MS (Figure 2).

## 4. Known Unknowns

There are unfortunately many important neurological diseases for which there are little or no epidemiological data available from Finland. There are no data on the epidemiology of atypical parkinsonism or brain iron accumulation disorders in Finland. The only study reporting neurosarcoidosis in the country found it as a cause of acute transverse myelitis in southern Finland in three cases over a study period of some 6 million person years [50]. In the other hand, the situation is the same for the neighbouring countries, with no data available from Estonia, Sweden or Russia, and there are only some regional data on neurosarcoidosis (in 2008–2019, the mean prevalence was 2.7/100,000 in Vestland county) and atypical parkinsonism (an incidence rate of 1.8/100,000 in the Faroe islands in 1995–2005) from Norway [69,70]. Earlier studies reported an increase in Myasthenia Gravis (MG) prevalence from 2.3/100,000 in 1964 to 5.6/100,000 in 1976, and more recent data tentatively suggest it may even be as high as 29/100,000 currently [71,72,73]. Although this figure should be treated with caution, it fits rather well between the prevalence figures reported for neighbouring countries, Estonia (23.5/100,000) and Sweden (36.1/100,000), whereas the prevalence seems to be considerably lower in Norway (12.8/100,000) [74,75,76]. There are no data available from Russia.

Estimates of migraine prevalence in Finland (10.1% among women; 2.5% among men) date back to data from 1981 [77]. Interestingly, contemporary data also suggest that the disorder might be much more common (28% among women; 9% among men) in northern Finland [78]. Hospitalisation with a migraine as the main diagnosis has become more common, but this might be associated with stroke care as a migraine may mimic a stroke [79]. No epidemiological data on other primary headache disorders are available. The migraine prevalence is also unknown in Norway, whereas the prevalence of cluster headaches in the country has been reported to be 48.6/100,000 [80]. In Sweden, the 1-year prevalence of migraine was reported as 13.2 ± 1.9% (16.7% among women and 9.5% among men) two decades ago. Moreover, there were no geographical differences or those between urban and rural areas [81]. Recent survey data suggest that the prevalence of migraine is 17.7% in Tartu, Estonia, and 20.8% in a countrywide population-based random sample of Russians [82,83]. It seems clear that more and better studies on headache epidemiology are needed, not only in low- and middle-income countries.

Normal pressure hydrocephalus (NPH) is apparently not rare in Finland [84,85], but its incidence and prevalence remain unknown. Interestingly, a recent study from eastern Finland reported that a C9orf72 expansion was found in 1.6% of possible NPH patients, but none of the controls [86]. Considering the C9orf72 expansion findings concerning ALS, a regional comparison of NPH incidence data would be very interesting, especially if they are augmented via mutation screening. There are no data available from neighbouring countries, besides a study reporting NPH as the most common cause of hydrocephalus surgery in Swedish adults in 1996–1998 (incidence 1.6/100,000 annually) and considerable regional variation [87].

The only data on Kennedy’s disease shows it to be common on the western coast [88]. The question posed by the authors (“Is the syndrome underdiagnosed?”) therefore remains unanswered. Nationwide genetic sample data from the previous millennium suggested that the minimum prevalence of Charcot–Marie–Tooth Disease is 1.2/100,000 in Finland [89]. A recent study, on the other hand, reports a CMT prevalence of 34.6/100,000 in northern Ostrobothnia [90]. Further efforts to investigate the occurrence of this disease are clearly needed, as are studies that report the epidemiology of other causes of polyneuropathy in the Finnish population. There are no data available on the epidemiology of these conditions in Estonia and Russia, whereas in northern Sweden, the prevalence of CMT was 20.1/100,000 in 1991. A recent study investigating the prevalence of hereditary neuromuscular disorders in northern Norway reported the prevalence of CMT as 29.9/100,000 and that of Kennedy’s disease as 2.4/100,000 [89]. It is interesting that the prevalence of CMT, therefore, appears to be somewhat higher in norther Finland compared to that in the northern areas of neighbouring Nordic countries. However, considering the lack of data from Lapland in Finland and that the Swedish data are over three decades old, no conclusions, besides the need for more work on the subject, should be drawn. Indeed, the CMT prevalence has been reported to be 42.3/100,000 in south-eastern Akershus County in Norway, and unpublished data suggest that the prevalence of CMT in all of Norway is 34/100,000 [91,92].

The nationwide incidence of Creutzfeldt–Jakob disease (CJD) was 0.6/1,000,000 in 1974–1989 [93], and 1.36–1.44/1 000 000 in Southwestern Finland in 2007–2013 [94]. More information on time trends and regional disease occurrence are clearly needed and will hopefully be provided in Eurosurveillance data. Additionally, while the average incidence of encephalitis from 1967 to 1991 was 1.4/100,000 adults/year in the Helsinki area [95], there are no current or nationwide data available. Furthermore, there are no Finnish epidemiological data on autoimmune encephalitidites (although the result of 1.4/100,000 adults/year must have certainly included some) or paraneoplastic neurological disorders. Therefore, a lot of basic neuroepidemiological work remains to be conducted in Finland. Data from 1985–1992 showed an incidence of 0.12/100,000 for CJD in Sweden [96], but otherwise, neighbouring countries also apparently rely on international surveillance data. On the other hand, clinical research on encephalitidides, especially tick-borne encephalitis (TBE), has been much more active in Sweden and Norway.

## 5. Great Unknowns

Perhaps surprisingly, uncertainty also colours our understanding of the most common neurological disorders to a great degree. The incidence of epilepsy in adults appears to be very much related to age, with a markedly higher rate in people >60 years of age, among whom it increased during 1989–2019, compared to that of younger adults, among whom the incidence has declined during the same period [97]. The reasons for these trends are unknown. However, these results are derived from administrative registry data and should, therefore, be interpreted cautiously. The only study that has validated epilepsy diagnoses among adults investigated all persons with a suspected epilepsy in 1960–1979 in eastern Finland, reporting a mean annual incidence of 24/100,000 and age-specific incidence ratios that increased with advancing age. The prevalence of active epilepsy was 629/100,000. Both the prevalence and incidence were higher among males. Interestingly, while the age-specific prevalence of active epilepsy increased until 40–50 years of age, it declined in the oldest age groups [98]. More recently, comparatively high incidence figures for status epilepticus have been reported in the area [99].

The incidence of traumatic brain injuries (TBI) is slightly above the western European average in Finland, but the TBI mortality is relatively high, suggesting that the injuries may be more severe than they are elsewhere [100,101,102,103,104]. The epilepsy incidence trends among elderly people may also have partially resulted from the increasing incidence of traumatic brain injury in this population [102], whereas age does not appear to be an independent risk factor for epilepsy after an haemorrhagic stroke in Finnish patients [105,106]. Another possible clue lies in the observation that, although the incidence of gliomas remained stable at the international average level in 1990–2016 in Finland, the incidence increased in the oldest age group [107]. Moreover, the increasing incidence of and improving survival with glioblastoma might be of significance here [108]. Clearly, a thorough nationwide investigation, including currently absent regional data, into the current causes and disease courses of epilepsy among adults, and especially elderly people, would be valuable. The results would be useful, not only for public health purposes, but also clinically considering the particular challenges of care involved [109,110,111,112,113]. Interestingly, a similar largely unexplained development in age-specific epilepsy incidence trends has previously occurred in Denmark [114,115]. The study would, therefore, also be of potential international significance.

One major disease that might be related to the incidence trends of epilepsy is a stroke. Unfortunately, more epidemiological data are also needed here. It is well known that cardiovascular disorders are, and have long been, more common in eastern and northern parts of the country. Although the cardiovascular disease incidence has declined markedly in Finland and the gap has narrowed, a difference between the east and the west remains [15,116,117]. A similar difference in terms of stroke incidence has been previously documented between eastern and western Finland [118], followed by a declining incidence in both eastern and western areas, as well as central parts of the country, but with a remaining difference [119,120,121]. Nevertheless, recent data concerning Transient Ischemic Attacks (TIAs) from north Savonia suggest that ischemic strokes remain relatively common in eastern Finland [122]. Previous data also show the importance of cultural factors in stroke incidence, which are evident when Swedish-speaking and Finnish-speaking Finnish men are compared (the Swedish-speaking minority also shows a lower all-cause mortality) [123,124,125]. It is, therefore, unfortunate that current data on nationwide stroke incidence and the recent trends in Finland are not available, let alone valid and comprehensive epidemiological sex-, age- and region-specific data that include variables indicating socioeconomic status, cultural associations and modifiable stroke risk factors. The only exception is the subarachnoid haemorrhage (SAH), and these results underscore the importance of having up-to-date and epidemiological data: SAH does not appear to be exceptionally common in Finland after all [126]. Furthermore, its incidence is decreasing, along with smoking rates [127,128]. This, along with twin data [129], shows the importance of environmental factors in SAH risk and reducing incidence. Moreover, physical activity has been shown to be associated with the SAH risk [130], providing further possibilities for intervention. Regional data show the substantial variation in SAH incidence within the country (overall incidences of 10.2–10.4/100,000 in the eastern and northern parts of the country and 7.4–9.1/100,000 in the western and southern parts) [131], suggesting areas of maximum impact for public health interventions. Timely epidemiological data on other stroke disorders are, therefore, also needed, but robustness must be ensured [126].

**Figure 4:**
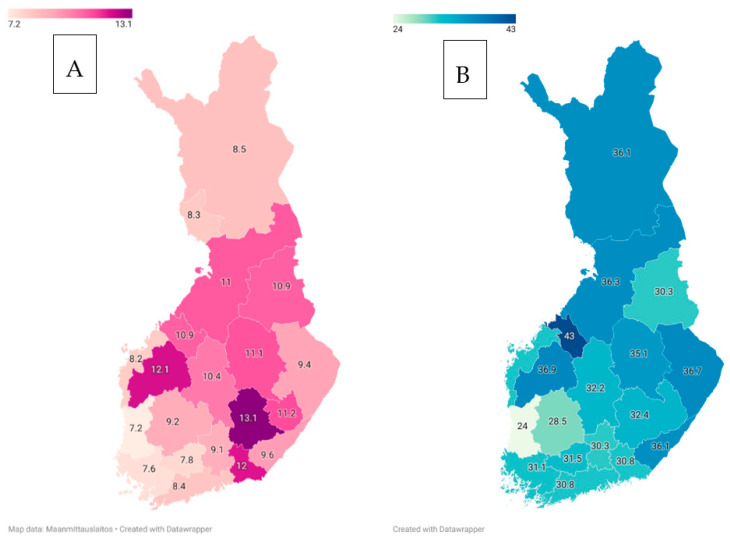
(**A**) The regional incidence (/100,000) of Alzheimer’s disease in Finland in 2007–2011. (**B**) The regional incidence (/100,000) of medicated parkinsonism in Finland in 2015–2019.

Information about vascular brain health is also needed to analyse its impact on trends in the epidemiology of Alzheimer’s disease (AD) [132]. Rather surprisingly, given the high quality and tradition of AD research in Finland [133,134,135,136,137,138], there are no current data on the incidence and prevalence of AD in Finland. The Current Care Guidelines report that there are 200,000 persons with mild cognitive impairment, 100,000 with mild dementia and 93,000 with at least moderate dementia in Finland [139]. These figures, however, are based on outdated data. More recent incidence figures can be estimated using MEDALZ data for 2005–2011, which mention that there are 294/100,000 people aged 30 years or more with AD, and 189/100,000 of the entire population have it [140,141]. However, this is an underestimate, and the actual figures are probably around 30% higher [142]. Using this information and MEDALZ data again to roughly estimate a median survival from five to eight years from diagnosis [143], the national AD prevalence cohort might currently be made up of around 70,000–120,000 people. The estimate remains very uncertain since elderly Finnish people are more physically and cognitively fit than they were just a few decades ago [144,145], and no useful data on AD incidence trends are available. Open data figures from The Social Insurance Institution of Finland (KELA) can be used to calculate a rough estimate (the same caveats apply [141]) of regional AD incidence in 2007–2011 (Figure 4) [146]. However, more robust data are clearly needed. Moreover, a coordinated investigation of the epidemiology and etiological background of both AD and NPH is warranted, considering the reported association between them [84]. There have been many studies concerning genetic factors that might predispose the Finnish population to AD. While these have not revealed any major culprits, it is worth knowing that amyloid-beta precursor protein (APP) duplications are not a common cause of early-onset AD in Finland [147,148].

Lastly, our knowledge about the epidemiology of Parkinson’s disease (PD) in Finland is not much more advanced than that of AD. Thorough investigations with diagnosis validation have only been made in south-western Finland, and the most recent one is now 30 years old. In the early 1970s, both the incidence and prevalence were relatively low and, interestingly, slightly higher in women compared to those of men [149]. This was estimated to be related to higher mortality from other causes among men. Two decades later, the incidence was slightly lower (14.7/100,000) than it previously had been among women and markedly higher (19.9/100,000) than it previously had been among men. The incidence had increased particularly among men over 70 years of age. Moreover, the disease was reported to be more common in rural areas than it was in urban areas [150]. Later data have reported that this rural–urban gradient seems to have remained unchanged [151]. However, all papers reporting data from the last 30 years rely on administrative data, with the diagnosis only being valid at the time it was made. The sensitivity and specificity of these data are therefore unknown, although a preliminary check suggested 80% accuracy [152]. It appears likely that these data (Figure 4) can be sufficiently relied upon to reach two conclusions: there is a ”belt” of higher PD incidence that runs diagonally across Finland [153], and the age-standardised overall incidence of the disease has remained unchanged for more than 25 years [154]. That last finding is similar to one recently reporting on neighbouring Estonia [155]. Only a handful of cases in which a gene variant has been associated with PD have been reported from Finland, and these have concerned GBA1, SNCA, RFC1 and PRKN [156,157,158,159,160,161]. Interestingly, most of the cases have been found in or have been related to eastern Finland. PD associated with GBA1, SNCA and RFC1 has been reported in north Karelia, where the author also diagnosed a family with POLG2-associated PD (POLG2-mutations have been observed also in ataxia patients in the region; all data are unpublished). Quite expectedly, LRRK2 does not seem to be associated with PD in Finland [162].

## 6. Perspectives and Pitfalls

Our knowledge about neuroepidemiology in Finland appears to be quite limited (Table 1), but some conclusions can, nevertheless, be drawn. Probably the most obvious ones are clinical: What we know about the occurrence of rare neurological disorders in Finland suggests that international guidelines and reviews are of limited use to Finnish clinicians pursuing a diagnosis of, for example, a hereditary ataxia disorder. This means that, since no national guidelines exist, unnecessary tests may be ordered and, on the other hand, useful tests may not be conducted. The situation is frustrating to clinicians and possibly even harmful to patients. Naturally, it is also costly, not just because of the long, dead-end diagnostic processes, but also because a specific diagnosis would aid in their tailoring care. National guidelines, such as the recent ones for the UK [163], are therefore needed, but it is unclear if we know enough to draft these. National efforts to better elucidate the landscape of rare neurological disorders would therefore benefit everyone.

Timely information about the prevalence and incidence trends of more common disorders would also be of practical help. These data would be of great help in assessing specialisation needs and designing care options for those with neurological disorders since there are obvious regional differences in these. Furthermore, to effectively combat, e.g., strokes, we need not only primary (and secondary), but also primordial preventions [165]. Epidemiological data are crucial to guide the implementation of these efforts and monitor the results. Excellent examples of the rewards yielded during epidemiological studies of this subject are the Finnish observations of the primacy of non-genetic factors in SAH risk, and especially, the role of smoking and its outsized relationship with females [127,128,129]. Since lifestyle factors influence the dementia risk in a modifiable way [135], primordial prevention efforts should also be designed with this in mind. Current epidemiological data on AD and other dementing disorders are therefore needed. On the other hand, these efforts should also be considered as a possibility to investigate the possible effects of these efforts on other disorders that are known to be influenced by environmental factors, such as AD, PD, MS and ALS. Moreover, granular nationwide data are needed to confirm or refute the suggested epidemiological links between these disorders [166,167].

These kinds of large-scale, prospective investigations of incidence trends would, therefore, also provide us with valuable information concerning the etiological factors of these disorders. As the currently available Finnish data show, there are considerable regional discrepancies in the epidemiology of many common neurological disorders, even in small populations such as the Finnish people. It therefore seems probable, that investigating, for example, so-called neurodegenerative disorders as one category at a national level may be misleading [166,168]. More granular data concerning the phenotype, area and time are therefore needed. Furthermore, since there are well-known problems concerning both the accuracy and reporting of epidemiological data [36,169,170], robust information can only be acquired via prospective planning. For example, the Finnish Care Registry for Healthcare (CRHC), while it is reliable in general, does have its pitfalls concerning accuracy and sensitivity, and these appear also to be diagnosis-specific: our CRHC search for 1987–2010 HD diagnostic codes identified 399 persons with one, but the subsequent chart review confirmed only 214 persons with HD [18]. Simple typing errors were very common, for example, the patient had actually been inebriated (F10) or had PD (G20), but the code had been erroneously punched in as G10. This was naturally compounded by the long study period during which one error was enough to add the person to the CRHC search results. On the other hand, virtually all patients who had a diagnostic code of G40.37 in CRHC were confirmed to actually have EPM1 [17]. Nevertheless, during a more recent experience concerning CRHC data on WD, 282 persons were identified in CRHC, of whom only 33 turned out to be WD patients [34], suggesting that CRHC data that have not been validated for a specific diagnosis should only be neuroepidemiologically used to investigate trends across time in clearly defined morbidities, not their actual incidence or prevalence figures. Although the problem clearly concerns also common disorders, such as stroke and AD [142,171], it is probably more pertinent when rare disorders are investigated since false positives (and negatives) have a much greater impact on the results in these studies. KELA medication reimbursement data can also be used to a certain extent to estimate the incidence and prevalence trends of PD and epilepsy. (These still require a detailed statement from a neurologist. The statement is checked and approved by specialist physicians at KELA before the right is granted. (Table 2).) However, the sensitivity of these data for AD is suboptimal [142] and only available up to 2012. The main problem with the ”PD” data is obviously that the initial diagnosis, upon which the reimbursement right is based, may be, and often is, erroneous [172]. Moreover, the right is also granted to patients with other levodopa-responsive disorders (although these can be differentiated at least to some extent using the diagnostic code in the statement). DMTs for MS are also reimbursed, but the annual numbers of new codes in the registry poorly correlate with the numbers of newly diagnosed patients reported from the same areas in epidemiological studies, although the correlation has improved lately [47].

Lastly, regional differences in neuroepidemiology also raise the question whether there may also be regional differences in disease phenotypes and prognoses. The author’s personal experiences, having practiced neurology both in south-western Finland and north Karelia, suggest that there are some at least concerning PD. The (nationally) high PD risk in north Karelia, together with the (relative) abundance (nationally) of genetic causes of PD associated with the province, do not suggest otherwise. It would be particularly interesting, considering the armamentarium of DMTs and uncertainty about which of them is optimal for a given patient [173], to investigate the possible discrepancies in phenotype and disease course between high-risk areas of south-western Ostrobothnia and south-western Finland and the (nationally) relatively low-risk area of north Karelia. However, concerns about data validity are again raised here since there are known weaknesses in MS scales [174]; so, even prospectively gathered real-world data have weaknesses.

It therefore appears that a lot more neuroepidemiological research is needed, but it will require resources and a concentrated effort. So far, most neuroepidemiology studies in Finland have been conducted in small projects, usually as a part of a Ph.D. thesis. Naturally, these could be coordinated to achieve some of the goals outlined previously. However, this might prove difficult and time-consuming due to problems related with the Act on the Secondary Use of Health and Social Data [175]. These projects would also be insufficient to gather the data needed to robustly monitor trends of the most common neurological disorders. Furthermore, conducting even smaller-scale studies that validate the diagnoses appears to be currently impossible; while a decade ago, it cost hundreds of euros to gather the patient charts of almost 400 people identified with an HD code in the CRHC, the hospital districts would at the end of 2022 charged well over 30,000 euros for submitting only the paediatric and adult neurology patient charts of 129 persons [176]. The validation of data was therefore cancelled, and it appears that code validation is currently impossible because of abortive pricing. The future of Finnish neuroepidemiological research, therefore, currently appears to be very uncertain, even bleak. If no corrective action is taken to ensure reasonable pricing and administrative processes, the only hope lies in national disease-specific registries, which gather data with in-built clinical validation. The national MS registry is currently the only neurological example of these available in Finland [177], but as its coverage shows, not all hospital districts (welfare areas from 1 January 2023 onwards) are willing or able to join them. The government should, therefore, ensure national funding for, and the implementation of the registries should be considered to be of primary significance. Nevertheless, this will leave the study of several neurological diseases out in the cold. Endeavours to enhance the validity of the national administrative data are therefore also needed.

## Figures and Tables

**Table 1:** Epidemiological characteristics of selected neurological diseases in Finland. Incidence and prevalence are presented as /100,000. AD, Alzheimer’s disease; ALS, Amyotrophic Lateral Sclerosis; EPM1, Unverricht-Lundborg Disease; FRDA, Friedreich’s Ataxia; GBS, Guilain-Barré syndrome; HD, Huntington’s Disease; IS, Ischemic Stroke; MS, Multiple Sclerosis; n.a., not applicable; SAH, Subarachnoidal Haemorrhage; TBI, Traumatic Brain Injury; SCA3, Machado–Joseph disease; WD, Wilson’s Disease; ?, unknown; ↑, more common; ↔, as common; ↓, less common. References: [15,17,18,19,20,21,22,36,37,38,48,58,59,60,131,141,146,154,164].

Disorder	Approximate Incidence	Approximate Prevalence	Substantial within-Country Variation	Compared to Western European Populations
MS	8–12	150–300	Yes	↑
GBS	1	?	?	↔
ALS	5	11	Yes	↑
AD	?	?	?	?
PD	45	300?	Yes	↔
IS	?	?	Yes	↑
SAH	9	?	Yes	↑
TBI	350	610	?	↔
HD	0.2	1.5–2.5	No	↓
WD	0.02	0.5	No	↓
EPM1	0.02	1.5	No	↑
FRDA	0	0	n.a.	↓
SCA3	0	0	n.a.	↓

**Table 2:** The total number of persons in Finland with a reimbursement right for selected chronic neurological conditions at the end of the year, 2011.

Disorder	N
Parkinson’s disease and comparable movement disorders	16,400
Epilepsy	60,650
Alzheimer’s disease	61,165

## Data Availability

All data are accessible through open sources which can be provided by the author upon request.
